# Some Varieties of Acute Intestinal Obstruction

**Published:** 1909-10-30

**Authors:** 


### Surgery.

### SOME VARIETIES OF ACUTE INTESTINAL OBSTRUCTION.

Intestinal obstruction in its acute form is the result of various conditions. In some of these the diagnosis is easy, as in the case of a strangulated hernia. Here the presence of an abnormal swelling, old or recent, calls attention to the *fons et origo* of the malady; or, at any rate, it should do so, for the first thing which should be examined in a case of sudden intestinal obstruction or of unexplained vomiting is each hernial orifice. It is not sufficient to rely upon the belief that, if the patient has a hernia, he will know this and mention it. Some patients are ruptured without knowing it; and others, who are fully aware that they possess a hernia, make no mention of the fact because they do not think it can be connected with their present symptoms. When a hernia comes down for the first time and is immediately strangulated, they are likely to call attention to it, because they have pain in the swelling. But it is more usual for strangulation to occur by the subsequent descent of a fresh coil of gut into a hernial sac of old standing, and these are the cases in which no mention is made of the presence of a hernia by the patient.

But where acute intestinal obstruction is due to causes other than the strangulation of hernia, the diagnosis is much more difficult. Whatever the cause may be, however, the clinical picture is more or less similar. The illness starts with the sudden onset of acute abdominal pain, referred usually to the umbilicus. This is accompanied by vomiting. This initial emesis is due, not to any mechanical action of the obstruction, but to shock following the interference with the splanchnic ganglia. The abdomen is quite flaccid at first, the pulse small and slow, and the temperature is subnormal. These are the symptoms of the stage of onset.

Later, and this is only a matter of hours, the typical picture develops. Vomiting sets in again and from now on is persistent, at first gastric, then bilious, and finally stercoraceous. The temperature remains subnormal, but the pulse becomes quick and irregular. The abdomen rapidly becomes distended and hyperresonant. This is due almost entirely to the liberation of gas in the coils; the damage to the vascular supply of the affected gut leads to an interference with the normal power of absorption of the capillary vessels. This distension is sometimes known as meteorism. Two distinct forms are recognisable, one in which there is a general distension of the abdomen, and another in which the distension is, more or less, localised to start with. This is due to obstruction of a single loop, as in a volvulus; it takes less time to develop than does the general distension.

It is not, of course, difficult to diagnose the presence of acute intestinal obstruction. What is difficult in any given case is to be able to say what is the cause and in what part of the alimentary canal the obstruction is situated. Immediate laparotomy is, of course, demanded in all cases; but for the actual position of the incision by which the abdomen should be opened, no hard and fast rule can be laid down. One variety of acute intestinal obstruction, namely intussusception, stands somewhat apart. Occurring, as it does nearly always in small children, and presenting symptoms characteristic of no other condition, the presence of blood and mucus in the rectum and of an oblong swelling palpable through the abdominal wall, it should be readily diagnosed. The best incision for these cases is a vertical one through the right rectus, with its centre opposite the umbilicus. The great majority of intussusceptions start in the neighbourhood of the cæcum, and this incision thus gives an easy access to the affected part of the intestine; but even if this is not the case, the incision can be enlarged upwards or downwards and the whole peritoneal cavity can be explored with moderate facility in a small child.

As a general rule the same incision will also be found best in adults. It is, at any rate, better than an incision in the middle line, since here the umbilicus is inconvenient if it is found necessary to enlarge the incisions, and, further, a sounder wound is likely to result when the muscle is divided than when the incision is made through the linea alba.

Of course, if the distension is confined to one quadrant of the abdomen, as it is in the earlier stages of a volvulus of the sigmoid, it is better to make an appropriate incision directly over the swelling.

The moment the parietal peritoneum is opened the distended intestines extrude themselves, and the next problem is to find the site of the obstruction. If it is possible to palpate the cæcum, its condition will form a valuable guide to the site of the obstruction; *e.g.* a distended cæcum will indicate that the obstruction is on the colon, and a collapsed one that the obstruction is in the small intestine. If this cannot be done, the coils of intestine should be brought carefully out of the wound into warm sterilised towels, and the gut should then be examined *seriatim* for the site of the obstruction.

We are not concerned here with the detailed treatment of the various conditions which may be found, such as strangulation by band or by a Mechel's diverticulum, strangulated hernia into a peritoneal fosse or volvulus, but only with some general considerations of the operative treatment. Suffice it to say that the obstruction must be relieved, and that if, in the opinion of the operator, the intestine is so damaged that it cannot survive, the damaged portion should be excised. But we would point out the extreme value of the process of tapping the distended coils by puncture with a scalpel and allowing their contents to escape before doing anything else. This can easily be done without contaminating the peritoneum, and the puncture can be sewn up with Lembert sutures. When the intestines have been emptied in this way, the subsequent difficulties of the operation are enormously decreased, and manipulations which are practically impossible, when dealing with acutely distended gut, become comparatively simple.

